# Subtrochanteric valgus osteotomy in nonunited femoral neck fractures in children: two different fixation methods

**DOI:** 10.1186/s13018-025-06051-0

**Published:** 2025-07-11

**Authors:** Mahmoud Badawy, Sami Ibrahim Sadek, Ahmed Mohamed Abdelwahab, Ibrahim Abdellatif Algohiny

**Affiliations:** 1 Zagazig University, Menia El-Qamh, Sharkia, 44111 Egypt; 2 Zagazig University, Zagazig, 7510001 Egypt; 3 Zagazig University, Mit Ghamr, 7510002 Egypt

**Keywords:** Pediatric neck femur fracture, Wagner technique, Nonunited femoral neck fracture, Subtrochanteric valgus osteotomy

## Abstract

**Introduction:**

Nonunion of pediatric femoral neck fractures is a difficult situation for orthopedic surgeons. It can result in devastating results if left untreated. The purpose of the present study was to assess and compare the results of the treatment of nonunited pediatric femoral neck fractures by closed reduction and subtrochanteric valgus osteotomy with two different fixation methods.

**Patients and methods:**

Seventeen patients with nonunion femoral neck fractures were treated with closed reduction, subtrochanteric osteotomy and fixation with either the proximal humerus internal locking system (PHILOS plate) (group A) (10 patients) or the Wagner technique (group B) (7 patients). The mean age of the patients was 7 years, and the mean follow-up period was 16 months.

**Results:**

At the end of the follow-up, all patients, the fractures were united. We did not observe any cases of implant failure. There was a statistically significant improvement in the neck shaft angle in both groups from 88.70 ± 3.36 to 129.35 ± 7.34 in group A and from 87 ± 1.91 to 126.78 ± 4.06 in group B. Regarding the clinical outcome and according to the Ratliff concept, half of the cases were good in group A and 57.1% were good in group B. There was no statistically significant difference between the groups regarding time to union, degree of coxa vara correction or the Ratliff concept.

**Conclusion:**

Both the PHILOS plate and the Wagner technique offer good stable fixation options for nonunited pediatric femoral neck fractures.

## Introduction

Femoral neck fracture in children is rare. It accounts for less than 1% of all pediatric fractures [1]. Many complications can result from this fracture including avascular necrosis, fracture nonunion, malunion, proximal femoral growth arrest and coxa vara [2]. 

The nonunion of the femoral neck fracture in children is not uncommon. It is the third most common complication after the avascular necrosis and coxa vara [2]. The nonunion rate is 0–44% depending on the location of the femoral neck fracture [3–5]. 

Subtrochanteric or intertrochanteric femoral valgus osteotomy with or without a bone graft is considered the mainstay of treatment of femoral neck fracture nonunion. However, the optimal fixation method for the osteotomy and the femoral neck nonunion is still debatable [2, 6, 7]. 

Various fixation methods were used, including the dynamic hip screw (DHS), pediatric locking compression plate (LCP), and blade plate, which are expensive [2, 7]. Sharma et al. [8] used two cancellous screws and a Kirschner wire (K-wire) in their case report study. A contoured dynamic compression plate (DCP) was also used but it allows only two screws through the femoral neck [9]. We introduced the use of both the proximal humerus internal locking system (PHILOS plate) and Wagner technique [10] (wire based blade plate) as cheap easily available fixation methods for nonunited pediatric femoral neck fracture treated by subtrochanteric valgus osteotomy.

## Patients and methods

Seventeen patients with nonunion pediatric femoral neck fractures underwent subtrochanteric valgus osteotomy with one of two different fixation methods. We conducted this retrospective study between January 2019 and May 2023. Ten patients (7 boys and 3 girls) were fixed by the proximal humerus internal locking system (PHILOS plate) manufactured by Synthes which is now a part of DePuy\ Synthes in USA, and 7 patients (4 males and 3 females) were fixed by the Wagner technique [10]. The choice of the Wagner technique was in cases with moderate to severe neck resorption. This study was approved by our Institutional Review Board (IRB), [Number, #10609\21\3\2023] and informed consent was signed by every child’s parents. The patients’ mean age was 7 (ranging from 5 to 10) years. According to the Delbert classification system popularized by Colonna [11], all the fractures were type II. The mean follow-up period was 16 (ranging from 12 to 24) months. The eligibility criteria included all traumatic nonunited pediatric femoral neck fractures even if complicated with avascular necrosis. While pathological pediatric femoral neck fracture nonunion (for example; bone cyst, osteogenesis imperfecta, and bone tumors) were excluded.

### Preoperative evaluation and planning

History, [Table 1] including time and the mechanism of initial trauma (low-energy trauma denoted bone softening disease), was documented. The time elapsed from injury to nonunion surgery, initial management and vitamin D deficiency patients were also noted.

**Table 1:** Patient demographics in both groups

Parameters	Group A		Group B	
	(*N* = 10)		(*N* = 7)	
	No.	%	No.	%
Age				
Range (years)	10-Jun		10-Jun	
Mean ± SD	7.80 ± 1.31		7.71 ± 1.49	
Side				
Right	5	50%	3	42.9%
left	5	50%	4	57.1%
Bone softening diseases				
Low vitamin D	4	40%	2	28.5%
Mechanism of initial trauma				
Road traffic accident	6	60%	4	57.1%
Fall from height	4	40%	3	42.9%
Initial management				
Hip spica	4	40%		
Cannulated screws	6	60%	5	71.4%
Dynamic hip screw	0		2	28.6%
time between the injury and the nonunion fixation				
Range (months)	10-Jun		9-May	
Mean ± SD	7.14 ± 1.29		7.80 ± 1.31	

Continuous variables were expressed as mean ± standard deviation (SD) & median (range), Categorical variables were expressed as number (percentage)

The clinical examination, included signs of bone softening disorders and the degree of femoral shortening was also documented.

**Fig. 1:**
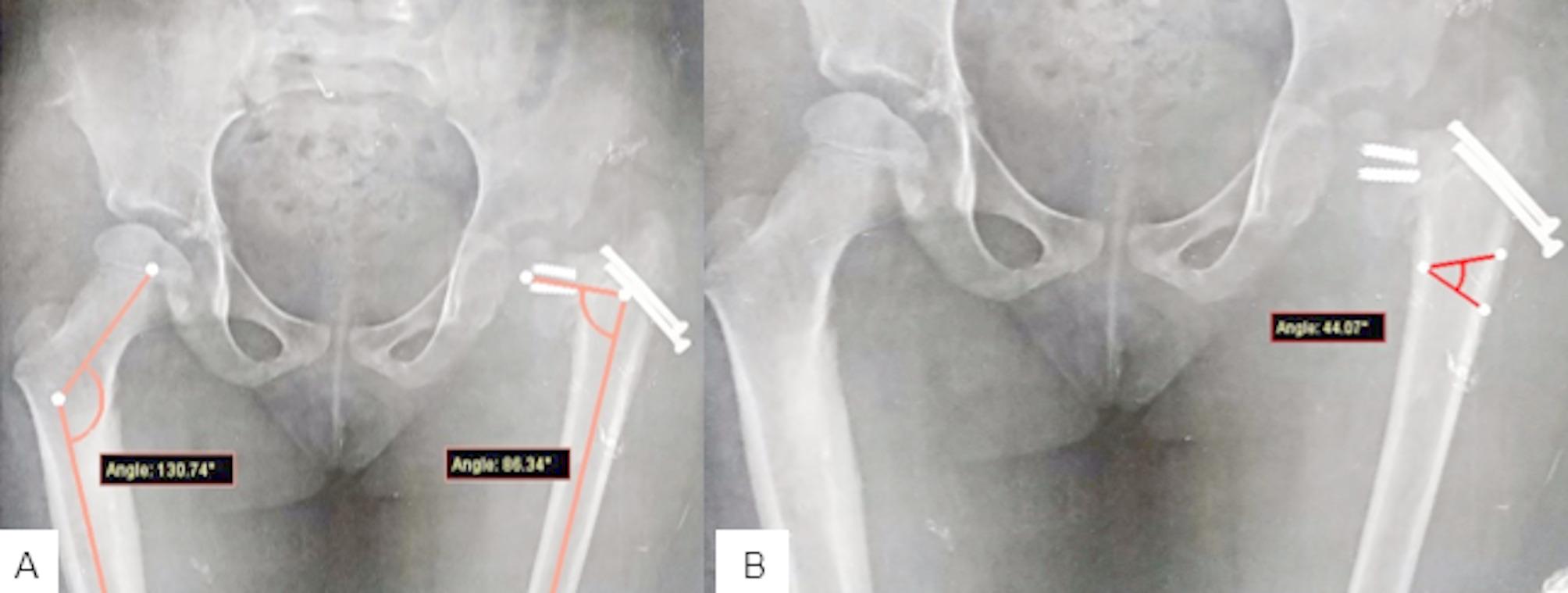
**A**; the difference in the neck shaft angle between the two sides was 44.070. **B**; this angle difference (44.07) was drawn in the subtrochanteric region to calculate the expected size of the valgus wedge

We measured the preoperative neck shaft angle of each patient and compared it to that on the normal side (Fig. 1A). The difference between the two angles was calculated, and a subtrochanteric wedge was drawn (Fig. 1B).

### Surgical technique

With the patient in the supine position and under general anesthesia, sterilization and draping of the affected lower extremity and pelvis were performed. A standard lateral approach to the femur was used. After the previous implants were extracted, the varus deformity at the fracture site was corrected by traction and abduction of the limb.

### Group A (PHILOS plate group)

**Fig. 2:**
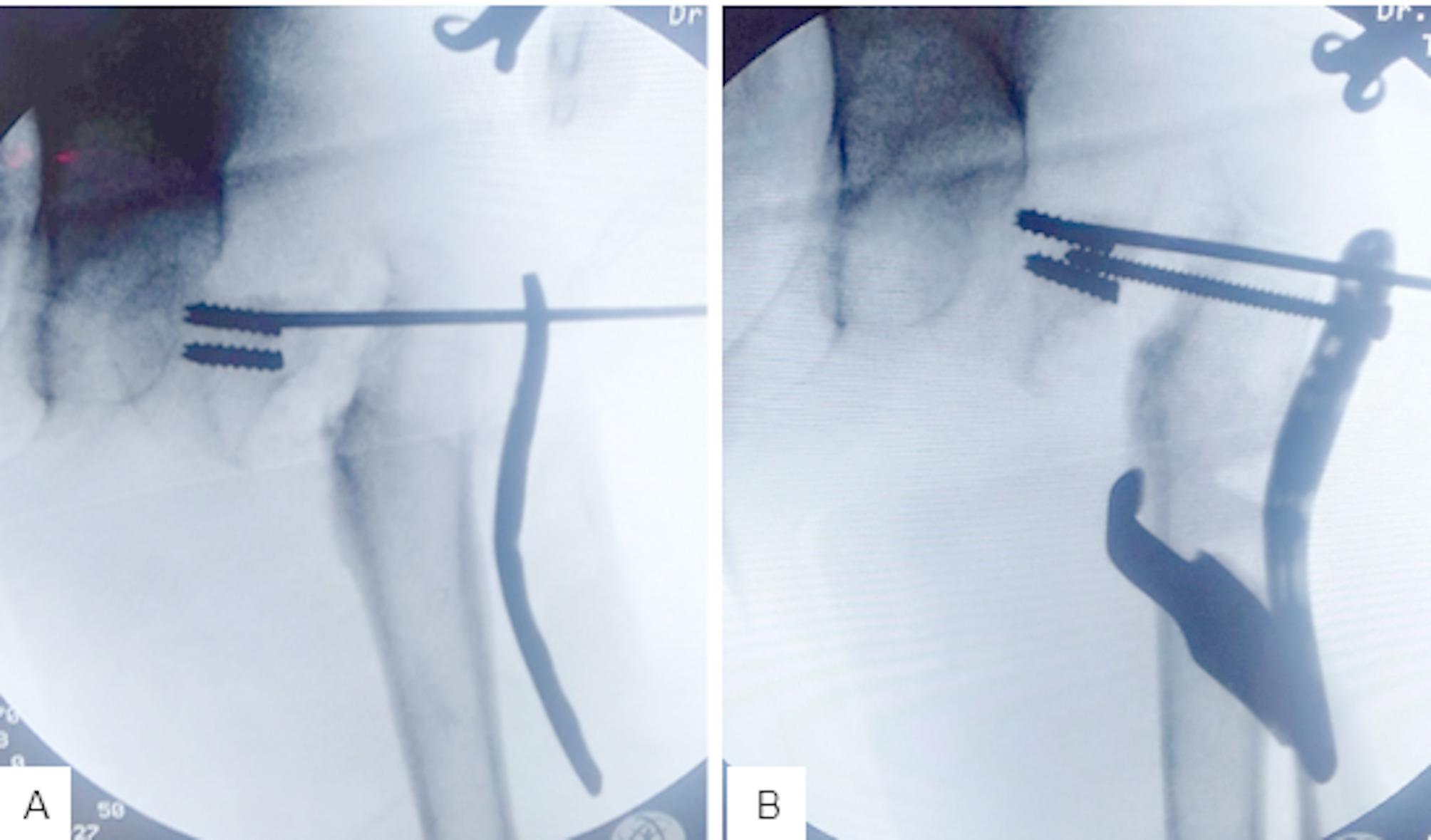
**A**; closed reduction of the nonunion femoral neck fracture and K-wire insertion at the superior neck. **B**; insertion of the superior screws and performing the subtrochanteric wedge

**Fig. 3:**
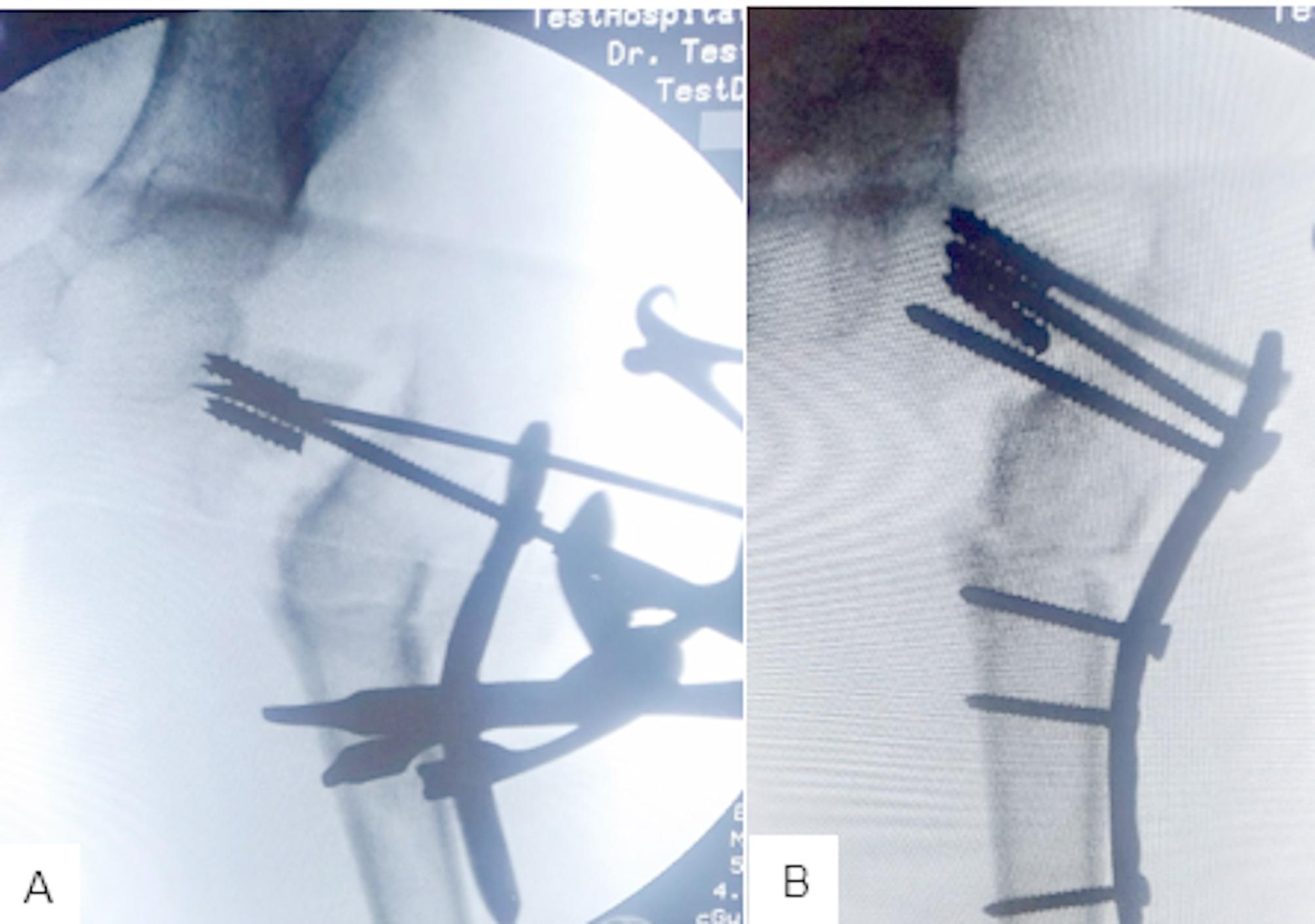
**A**; closure of the subtrochanteric osteotomy wedge. **B**; insertion of the proximal and distal screws

**Fig. 4:**
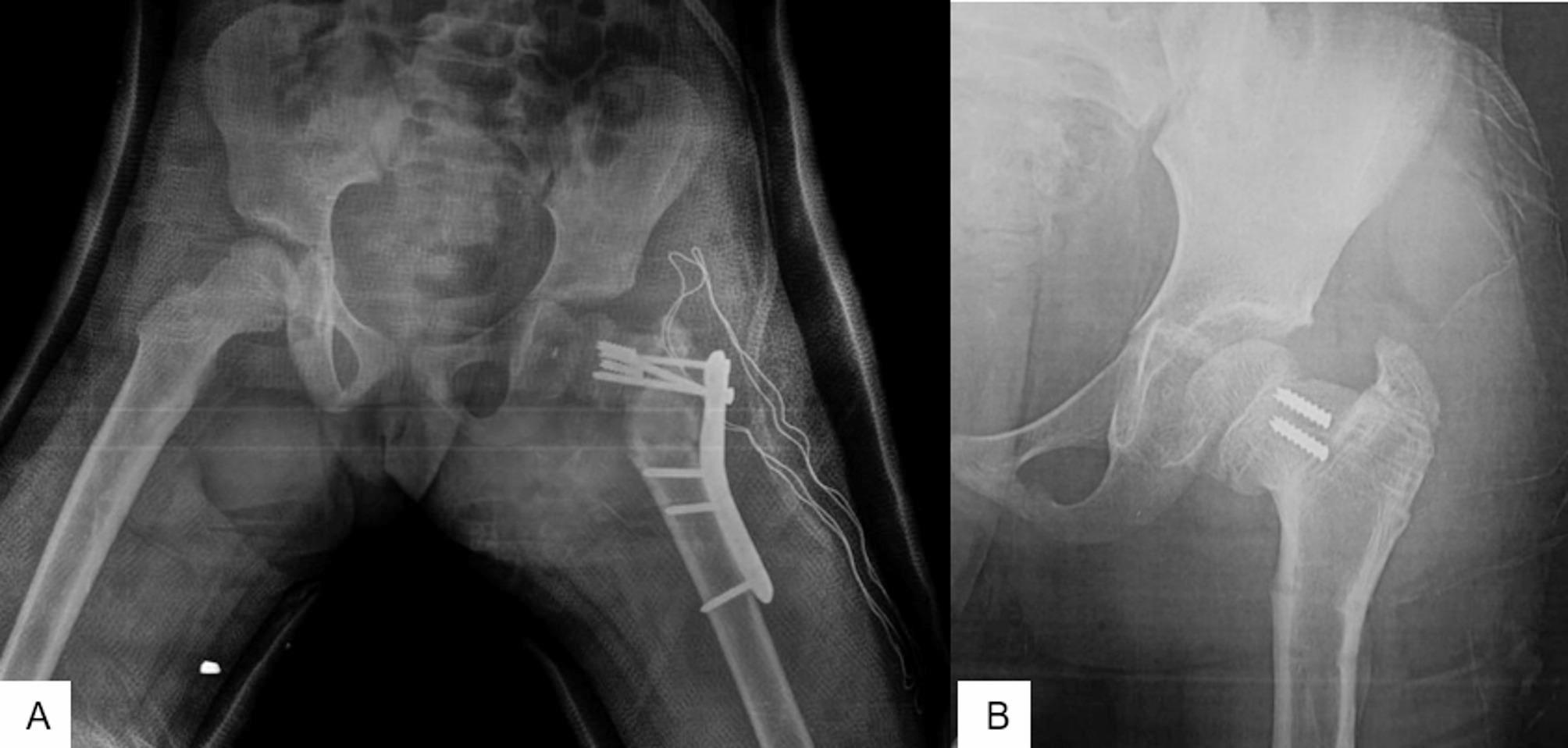
**A**; immediate postoperative follow-up of a 6-year-old girl with nonunion femoral neck fracture. **B**; final follow-up of the same case after plate extraction

Preliminary fixation of the reduced fractured neck femur was performed with a suitably sized K-wire [1.2–1.8 mm] through the PHILOS plate (Fig. 2A). The PHILOS plate was bent in valgus. Superior screws were inserted first into the superior neck. The subtrochanteric valgus osteotomy was then performed according to the preoperative plan (Fig. 2B). The size of the removed wedge was usually less than the calculated valgus angle, and we depended on the translation of the distal fragment for full correction of the deformity so as not to remove too much bone. Lateralization of the distal fragment was planned in all patients because of its mechanical advantages. However, this was not always possible. After closure of the wedge (Fig. 3A and B), distal screws and the remaining proximal screws were inserted. The neck shaft angle was measured to evaluate the correction. We then closed the wound in layers and applied a hip spica. Figure 4 shows the post-operative follow-up of a 6-year-old girl with nonunion of femoral neck fracture (Fig. 4A and B).

### Group B (Wagner technique)

**Fig. 5:**
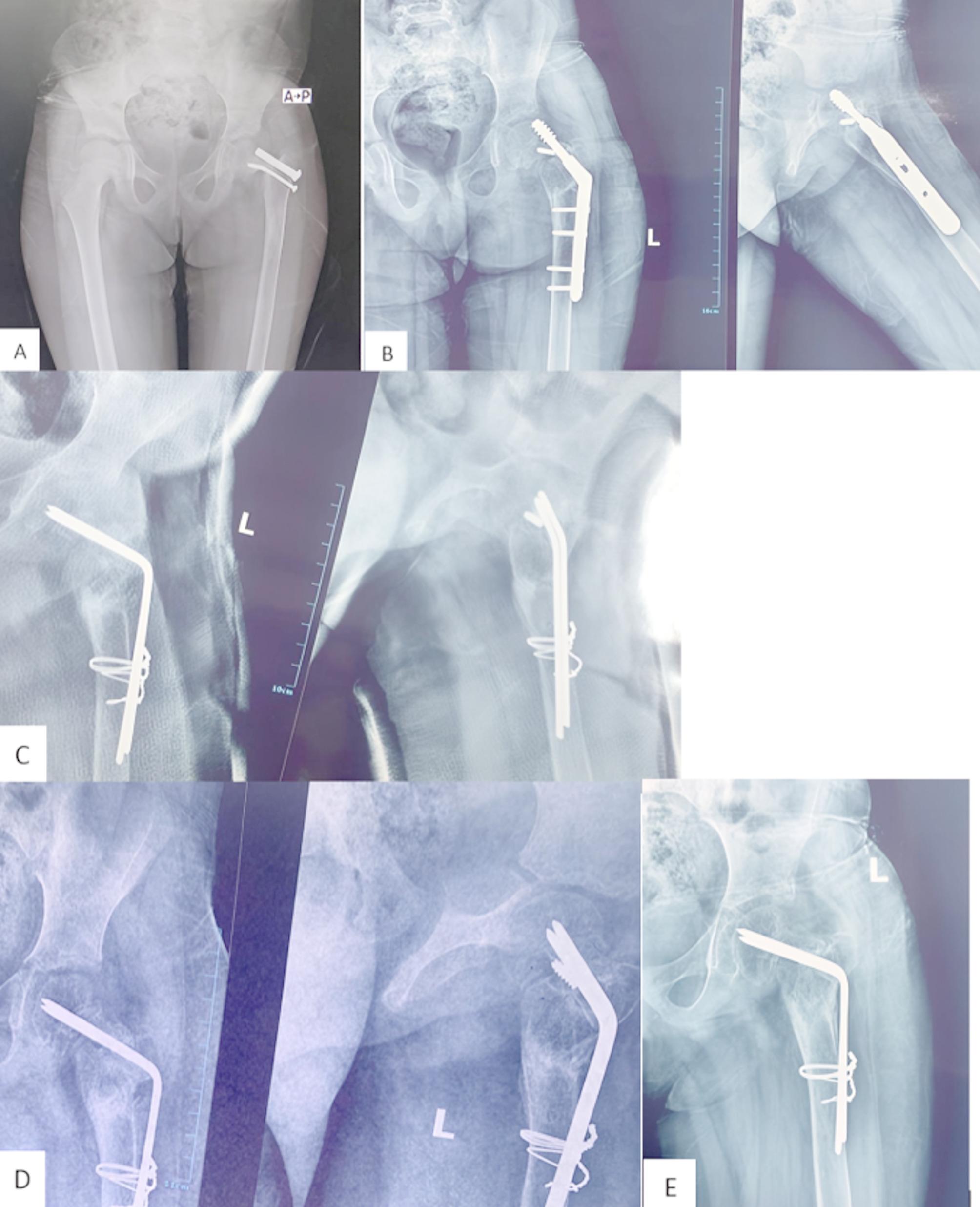
**A** 5-year-old girl with nonunited left femoral neck fracture and failed cannulated screws fixation. **B**: Six months later, she was then revised by a dynamic hip screw, that was complicated by a lag cut through and marked neck resorption. **C**, **D**: Finally, she was revised by removing the DHS and fixed by the Wagner technique with a fibular strut graft; **C**: early post-operative 4 months after surgery and **D**: Eight months after surgery. **E**: X-ray of the left hip shows united femoral neck fracture and united subtrochanteric osteotomy

Wagner technique [10] is based on the insertion of multiple K-wires into the femoral neck passing through the physis to the subchondral bone of the femoral head. These wires are subsequently bent at the lateral femoral cortex to be fixed to the femoral shaft via two cerclage wires. We inserted 2–3 thick K-wires [2.5–3 mm] into the femoral neck to reach the subchondral bone of the femoral head. Sutrochanteric valgus osteotomy was then performed according to the preoperative plan. The wires were subsequently bent at the lateral femoral cortex to be fixed to the femoral shaft via multiple cerclage wires. The cerclage wires were inserted either through a wire passer subperiosteally around the femur or through holes at the center from the anterior to posterior surface of the femur. In three patients, we used an autologous ipsilateral fibular strut graft; in these patients, there was marked resorption of the femoral neck. The fibular graft was either inserted through the tunnel of the lag of the pediatric DHS in revision cases [2 cases] or through a 7 mm drill pit [one case]. Figure 5 shows a failed revision nonunion femoral neck fracture operated by a pediatric DHS. She was then revised by Wagner technique (Fig. 5).

### Postoperative and follow-up

We preferred to apply a hip spica for all our patients for 6 weeks, because all of them are 10 years old or less, to protect our fixation in this young age group. Antero-posterior plain X-ray view of the pelvis was obtained in the immediate postoperative period to assess the accuracy of fracture reduction. Then, every four weeks until fracture union, then at the final follow-up. Clinical outcomes were assessed according to the Ratliff concept [6].

Implant removal was routinely done one and a half years after the revision surgery after the solid union of both the femoral neck nonunion and the subtrochanteric osteotomy.

### Statistical analysis

All the data were collected, tabulated and statistically analyzed via Microsoft Office Excel 2010 for Windows (Microsoft Cor., Redmond, WA, USA) and SPSS 22.0 for Windows (IBM Inc., Chicago, IL, USA). Continuous quantitative variables are expressed as the means ± SDs and medians (ranges), and categorical qualitative variables are expressed as absolute frequencies (numbers) and relative frequencies (percentages). Continuous data were checked for normality via the Shapiro‒Wilk test. The Mann‒Whitney U test was used to compare two groups of non-normally distributed variables. The Wilcoxon signed-rank test was used to compare two dependent groups of non-normally distributed data. The percentages of categorical variables were compared via Pearson’s chi-square test or Fisher’s exact test when appropriate. All tests were two sided. A P value < 0.05 was considered statistically significant (S), a p value < 0.001 was considered highly statistically significant (HS), and a p value ≥ 0.05 was considered statistically insignificant (NS).

## Results

At the end of the follow-up period, all femoral neck fractures were united. The union period ranged from 2.5 to 3 months, with no statistically significant difference between the groups. There was a statistically significant improvement in the neck shaft angle in both groups from 88.70 ± 3.36 to 129.35 ± 7.34 in group A and from 87 ± 1.91 to 126.78 ± 4.06 in group B. There was no statistically significant difference between the groups with respect to the degree of correction of the neck shaft angle. Regarding the clinical outcome; according to the Ratliff concept [6]there were no bad results, with half of the cases being good in group A and 57.1% being good in group B. There was no statistically significant difference in the Ratliff concept between the two groups. Gender has no statistically significant impact on the final Ratliff concept. 50% of the girls were good while 45.5% of the boys were good [Table 2].

We had 9 cases of fair results of Ratliff concept [6] with mild avascular necrosis at the final follow-up but because of the short-term follow-up, we cannot totally exclude the possible late appearance of more cases. Also, we didn’t have any cases of implant failure or implant breakage during removal.

**Table 2:** Comparison between groups A and B regarding neck shaft angle and Ratliff concept

Parameters	Group A (*N* = 10)		Group B (*N* = 7)		Test	*p*-value (Sig.)
	No.	%	No.	%		
Neck shaft angle preoperative (^0^)						
Mean ± SD	88.70 ± 3.36		87 ± 1.91		-0.992a	0.321
Median (Range)	88 (85–95)		87 (85–90)			(NS)
Neck shaft angle final follow-up (^0^)						
Mean ± SD	129.35 ± 7.34		126.78 ± 4.06		-1.183a	0.237
Median (Range)	130.25 (115–138)		129 (121–135.50)			(NS)
Testb	-2.805		-2.366			
*p*-value (Sig.)	0.005 (S)		0.018 (S)			
Time of union (months)						
Mean ± SD	2.70 ± 0.25		2.71 ± 0.26		-0.114a	0.909
Median (Range)	2.50 (2.50–3)		2.50 (2.50–3)			(NS)
Ratliff consept						
Fair	5	50%	3	42.9%	0.084c	1.000
Boys	3	60%	2	66.7%		
Girls	2	40%	1	33.3%		
Good	5	50%	4	57.1%		(NS)
Boys	4	80%	2	50%		
Girls	1	20%	2	50%		

Continuous variables were expressed as mean ± SD & median (range), Categorical variables were expressed as number (percentage); a: Mann Whitney U test; b: Wilcoxon signed ranks test; c: Chi-square test; *p*-value < 0.05 is significant; Sig.: Significance

## Discussion

Although rare, nonunion of the femoral neck fracture is a formidable complication if left untreated. Many factors can cause nonunion of femoral neck fractures, including non-anatomical fracture reduction, delayed presentation, inadequate fixation, fracture type, and associated pathology [12]. The most important cause of nonunion in developed countries is inadequate reduction or inadequate fixation; however, delayed presentation is the most common cause of nonunion in developing countries [2, 8]. 

Closed reduction and intertrochanteric valgus osteotomy with or without bone grafts are the mainstay of treatment for nonunited femoral neck fractures. Closed reduction helps correct the varus deformity at the fracture site and increases the bony contact between the ends of the fracture fragments. Open reduction is usually not needed, as it may affect the vascularity of the femoral head. Additionally, fibrous union at the fracture site is usually transformed into bony union after the correction of biomechanical defects [9, 13, 14]. Damany and colleagues [15] reported that the rate of nonunion after open reduction of the femoral neck was 11.2%, whereas it was 4.7% after closed reduction.

Intertrochanteric or subtrochanteric osteotomy has the advantage of transforming the shearing forces at the fracture site into compression forces that are biomechanically sound for fracture healing. It also helps in the correction of coxa vara deformities associated with nonunion femoral neck fractures [7, 16]. 

The use of bone grafts in nonunited femoral neck fractures is debatable, but generally speaking, bone grafts are important for cases with femoral neck resorption. Bone grafting involves either nonvascularized or vascularized bone grafts. Bone grafting has the advantages of stimulating bone healing, filling the bone defect, and adding stability at the fracture site [17]. Elgeidi and El-Negery [9] used a nonvascularized fibular strut graft alone for both fixation of the fracture site and enhancement of bone healing. Vascularized bone grafts help revascularization of the femoral head, but they are technically difficult and require specialized centers [18]. The presence of avascular necrosis of the femoral head usually does not alter the management plan for nonunited pediatric femoral neck fractures. Revascularization of the femoral head was noted after the union of femoral neck fractures [19]. 

Many fixation methods, such as a pediatric dynamic hip screw, blade plate, or plates and screws, have been proposed for fixation of femoral neck fractures with subtrochanteric osteotomy. Each of these has advantages and disadvantages. The dynamic hip screw and fixed angle blade plate are relatively expensive, needs adequate bone stock in the femoral neck to allow rigid fixation, and cannot pass through the physis [6, 8, 9]. Sharma and colleagues [8] used two cancellous screws and a K-wire in their case report study for fixation of the nonunited neck femur fracture. Only a single toddler case was the study population that is difficult to popularize to older children. Elgeidi and El-Negery [9] used a contoured dynamic compression plate for fixation, but it only permits two screws fixation through the femoral neck [9]. 

To our knowledge, this is the first study that uses both the Wagner technique and the PHILOS plate to fix a nonunited pediatric femoral neck fracture with subtrochanteric osteotomy. Both implants are inexpensive, easily available in most centers, and represent a good alternative to expensive implants, especially in developing countries. PHILOS plates have the advantages of allowing the insertion of multiple screws at the fracture site, being relatively cheap, being able to be bent, allowing adjustable correction of varus deformities, and having a suitable contour covering the pediatric proximal femur.

The Wagner technique was first introduced by Wagner 1978 for fixation of intertrochanteric osteotomy for coxa vara correction [10]. It represented a wire based blade plate. It was then used for fixation of pediatric proximal femurs in different pathologies, such as bone cysts, femoral neck fractures and fibrous dysplasia [20–22]. We used this technique in the present study for nonunited femoral neck fractures with various degrees of neck resorption. The advantages of this technique include stable fixation even with neck resorption, as the K-wires can pass through the physis to engage the subchondral bone of the femoral head. Additionally, it allows adjustable correction of the varus deformity via adjustable bending of the K-wires. However, because of its limited rigidity, it requires additional fixation by a hip spica. Additionally, there is a lack of standardization of the number and size of the K-wires.

All patients in the current study were united at the final follow-up, and the mean time to union was 2.7 and 2.71 months in both groups. There was no statistically significant difference between the groups in terms of the time to union, degree of coxa vara correction or Ratliff concept. Our results compared to other studies are presented in Table 3.

**Table 3:** Comparison between our results and other similar studies

Study	Number of cases	Mean age (years)	Delbert classification	Management	Follow-up (months)	Union time (weeks)	Result	Complications
Magu et al. [7] (2007)	10	10.19	All type II	Intertrochanteric Valgus osteotomy (Pauwel osteotomy) fixed with blade plate	98.4	16.6	8 good and 2 fair	Premature epiphyseal closure Two patients had 1–1.5 cm shortening
Neto et al. [23 (2008)	9	10.18	5 type II 4 type III	Subtrochanteric valgus osteotomy with fixed angle plate fixation	38	10.9	3 good, 4 fair, and 1 poor	Redo surgery in one patient Late osteonecrosis in one patient
Elgeidi and El-Negery. [9] (2017)	12	8.2	All type 2	6 cases had fibular strut graft and subtrochanteric valgus osteotomy with contoured plate (narrow DCP) 6 cases had fibular graft only and no osteotomy	20.4	14	11 good and 1 fair	Non
Nagi et al. [24] (1992)	17	13.35	10 type II 7 type III	Open reduction and internal fixation with 1 cancellous lag screw and free fibular graft	48.1	-	13 good, 3 fair, and 1 poor	one new case of avascular necrosis Four cases had coxa vara, and four cases had premature epiphyseal closure
Current study	17	7	All type II	Closed reduction and subtrochanteric osteotomy fixed in 10 cases with PHILOS plate and 7 case fixed by Wagner technique	16	10.8	9 good, and 8 fair	Non

## Conclusion

Both the Wagner technique and the PHILOS plate are good fixation methods for the treatment of nonunited femoral neck fractures with subtrochanteric osteotomy. There was no statistically significant difference between the two fixation methods in terms of the union rate, union time or Ratliff concept.

**Limitations of the current study** include the small sample size in each group, this may be attributed to the rarity of the nonunion of the pediatric femoral neck fractures. Also, the current study gives a short term results so late complications like osteonecrosis, and leg length discrepancy cannot be well estimated, and a longer term follow-up study is needed in the future to accurately evaluate possible late complications.

## Data Availability

No datasets were generated or analysed during the current study.
